# In Vitro Studies on the Relationship Between the Antioxidant Activities of Some Berry Extracts and Their Binding Properties to Serum Albumin

**DOI:** 10.1007/s12010-013-0712-2

**Published:** 2014-01-22

**Authors:** Jacek Namiesnik, Kann Vearasilp, Alina Nemirovski, Hanna Leontowicz, Maria Leontowicz, Pawel Pasko, Alma Leticia Martinez-Ayala, Gustavo A. González-Aguilar, Milan Suhaj, Shela Gorinstein

**Affiliations:** 1 Department of Analytical Chemistry, Chemical Faculty, Gdańsk University of Technology, 80952 Gdańsk, Poland; 2 Faculty of Pharmacy, Srinakharinwirot University, Bangkok, Thailand; 3 The Institute for Drug Research, School of Pharmacy, Hadassah Medical School, The Hebrew University, Jerusalem, 91120 Israel; 4 Department of Physiological Sciences, Faculty of Veterinary Medicine, Warsaw University of Life Sciences (SGGW), Warsaw, Poland; 5 Department of Food Chemistry and Nutrition, Medical College, Jagiellonian University, 9 Medyczna Street, 30-688 Krakow, Poland; 6 Centro de Desarrollo de Productos Bioticos, Instituto Politécnico Nacional, Carretera Yautepec-Jojutla, Km. 6, calle CEPROBI No. 8, Col. San Isidro, Yautepec, Morelos 62731 México; 7 Research Center for Food & Development, A.C. (CIAD), Carretera a Ejido La Victoria, Km 0.6, Hermosillo, Sonora 83304 Mexico; 8 Food Research Institute, 82475 Bratislava, Slovakia

**Keywords:** Berries, Bioactive compounds, Antioxidant activity, Binding properties

## Abstract

The aim of this study was to investigate the possibility to use the bioactive components from cape gooseberry (*Physalis peruviana*), blueberry (*Vaccinium corymbosum*), and cranberry (*Vaccinium macrocarpon*) extracts as a novel source against oxidation in food supplementation. The quantitative analysis of bioactive compounds (polyphenols, flavonoids, flavanols, carotenoids, and chlorophyll) was based on radical scavenging spectrophometric assays and mass spectrometry. The total phenolic content was the highest (*P* < 0.05) in water extract of blueberries (46.6 ± 4.2 mg GAE/g DW). The highest antioxidant activities by 2,2-diphenyl-*1*-picrylhydrazyl radical scavenging assay and Cupric reducing antioxidant capacity were in water extracts of blueberries, showing 108.1 ± 7.2 and 131.1 ± 9.6 μMTE/g DW with correlation coefficients of 0.9918 and 0.9925, and by β-carotene linoleate assay at 80.1 ± 6.6 % with correlation coefficient of 0.9909, respectively. The water extracts of berries exhibited high binding properties with human serum albumin in comparison with quercetin. In conclusion, the bioactive compounds from a relatively new source of gooseberries in comparison with blueberries and cranberries have the potential as food supplementation for human health. The antioxidant and binding activities of berries depend on their bioactive compounds.

## Introduction

It is well known that antioxidants present in various fruits, vegetables, juices, and wines have the potential to protect the urinary bladder, prevent cholesterol in blood, and protect the liver from free radical damage [1–3]. The various health benefits of berries are well documented and have been attributed mainly to their antioxidant capacity. There is a growing public interest for cranberry, blueberry, and relatively new gooseberry as a functional food because of the potential health benefits linked to phytochemical compounds [4] responsible for secondary plant metabolites (flavonols, flavan-3-ols, proanthocyanidins, and phenolic acid derivatives). Several different mechanisms have been proposed to explain the possible role of cranberries, blueberries, and gooseberries in the prevention of atherosclerosis [4–6].

Fractions responsible for the antioxidant action were identified and seem promising for phytomedicinal development [7]. Recent advances have been made in scientific understanding of how berries promote human health and prevent chronic illnesses such as some cancers, heart disease, and neurodegenerative diseases [8]. In fact, 90-day and 48-h stability of the blackberry extract in biologically relevant buffers has been investigated in studies [9]. Blackberry administration could minimize the toxic effects of fluoride, indicating its free radical scavenging and potent antioxidant activities. The induced oxidative stress and the alterations in antioxidant system were normalized by the oral administration of 1.6 g/kg body weight of blackberry juice [10]. Consumption of cranberries is known to exert positive health effects, especially against urinary tract infections. Cranberry was investigated as a chemotherapeutic agent [11]. For this reason, presumably, they are used in folk medicine [12]. *Physalis peruviana* (PP) is a widely used medicinal herb for treating cancer, malaria, asthma, hepatitis, dermatitis, and rheumatism [13–16]. Kusznierewicz et al. [17] analyzed different Polish cultivars of blue-berried honeysuckles and wild and bog bilberry for bioactive compounds. Potential benefits of polyphenolic compounds from raspberry seeds of three different extracts as efficient antioxidants were studied [18]. Infusions of *Ugni molinae* Turcz, also known as “Murtilla”, have long been used in traditional native herbal medicine [19] and investigated as well. However, the mechanisms behind the functions of berries with proteins are poorly understood. The interactions between polyphenols, especially flavonoids and plasma proteins, have attracted great interest among researchers. Few papers, however, have focused on the structure–affinity relationship of polyphenols on their affinities for plasma proteins [7, 20, 21], especially from berries. We were interested to investigate relatively new kind of cape gooseberries (*P. peruviana*) and to compare its composition with that of the widely consumed blueberries and cranberries. To meet this aim, the contents of bioactive compounds (polyphenols, flavonoids, flavanols, carotenoids, and chlorophylls) and the level of antioxidant activity (AA) were determined and compared. In order to receive reliable data, AA was determined by three assays: CUPRAC, DPPH, and β-carotene linoleate model system [22–24]. Human serum albumin is the drug carrier’s protein and serves to greatly amplify the capacity of plasma for transporting drugs. It is interesting to investigate in vitro how this protein interacts with flavonoids extracted from berry samples in order to get useful information of the properties of flavonoid–protein complex. Therefore, the functional properties of a new kind of berry will be studied by the interaction of water polyphenol extracts with a small protein such as HSA, using 3D-FL. As far as we know, no results of such investigations were published.

## Materials and Methods

### Chemicals

6-Hydroxy-2,5,7,8-tetramethylchroman-2-carboxylic acid (Trolox), 1,1-diphenyl-2-picrylhydrazyl (DPPH), β-carotene, linoleic acid, quercetin, human serum albumin, Tris, tris(hydroxymethy1)aminomethane, Folin–Ciocalteu reagent, lanthanum (III) chloride heptahydrate, CuCl_2_ · 2H_2_O, and 2,9-dimethyl-1,10-phenanthroline (neocuproine) were purchased from Sigma Chemical Co., St Louis, MO, USA. All reagents were of analytical grade. Deionized and distilled water was used throughout.

### Samples

Cape gooseberries (*P. peruviana*), blueberries (*Vaccinium corymbosum*), and cranberries (*Vaccinium macrocarpon*) were investigated. All berries were purchased at the local market in Gdansk and Warsaw, Poland. For the investigation, five replicates of five berries each were used. Their edible parts were prepared manually without using steel knives. The prepared berries were weighed, chopped, and homogenized under liquid nitrogen in a high-speed blender (Hamilton Beach Silex professional model) for 1 min. A weighed portion (50–100 g) was then lyophilized for 48 h (Virtis model 10-324), and the dry weight was determined. The samples were ground to pass through a 0.5-mm sieve and stored at −20 °C until the bioactive substances were analyzed.

### Extraction of Phenolic Compounds

The lyophilized samples of berries (1 g) were extracted with 100 mL of methanol/water (1:1) at room temperature and in darkness for 24 h. The extracts were filtered in a Buchner funnel. After removal of the methanol in a rotary evaporator at a temperature below 40 °C, the aqueous solution was extracted with diethyl ether and ethyl acetate, and then the remainder of the aqueous solution was freeze-dried. The organic fractions were dried and redissolved in methanol. These extracts were submitted to MS analysis for determination of bioactive compounds [25].

### Determination of Bioactive Compounds and Antioxidant Activities

The polyphenols were determined by Folin–Ciocalteu method with measurement at 750 nm with a spectrophotometer (Hewlett-Packard, model 8452A, Rockville, MD, USA). The results were expressed as mg of gallic acid equivalents (GAE) per g DW [26].

Flavonoids, extracted with 5 % NaNO_2_, 10 % AlCl_3_ · 6H_2_O, and 1 M NaOH, were measured at 510 nm. The total flavanols amount was estimated using the *p*-dimethylaminocinnamaldehyde (DMACA) method, and then the absorbance at 640 nm was read. To ensure the presence of flavanols on the nuclei, subsequent staining with the DMACA reagent resulted in an intense blue coloration in the plant extract [27]. As was mentioned previously, (+)-catechin served as a standard for flavonoids and flavanols, and the results were expressed as catechin equivalents (CE). Total chlorophyll, chlorophylls *a* and *b*, and total carotenoids were extracted with 100 % acetone and determined spectrophotometrically at different absorbances (nm) such as at 661.6, 644.8, and 470, respectively [28].

#### MS Analysis

A mass spectrometer, TSQ Quantum Access Max (Thermo Fisher Scientific, Basel, Switzerland), was used. Analytes were ionized by electrospray ionoization (ESI) in negative mode. Vaporizer temperature was kept at 100 °C. All samples were done by direct infusion in the mass spectrometer by using ESI source at negative ion mode, full scan analysis, ranging between 100 and 900 m/z. For optimization of the acquisition parameters and for identity confirmation, only a part of the standards was employed, not for all compounds that were found in the investigated samples. Settings for the ion source were as follows: spray voltage 3,000 V, sheath gas pressure 35 AU, ion sweep gas pressure 0 AU, auxiliary gas pressure at 30 AU, capillary temperature at 200 °C, and skimmer offset 0 V [29–31]. The AA was determined by the following assays:

1. Cupric reducing antioxidant capacity (CUPRAC): This assay is based on utilizing the copper (II)-neocuproine [Cu (II)-Nc] reagent as the chromogenic oxidizing agent. To the mixture of 1 ml of copper (II)-neocuproine and NH_4_Ac buffer solution, acidified and non-acidified methanol extracts of berry (or standard) solution (*x*, in ml) and H_2_O [(1.1 − *x*) ml] were added to make a final volume of 4.1 ml. The absorbance at 450 nm was recorded against a reagent blank [22].
2. Scavenging free radical potentials were tested in solution of 1,1-diphenyl-2-picrylhydrazyl (DPPH). In its radical form, DPPH has an absorption band at 515 nm which disappears upon reduction by an antiradical compound. DPPH solution (3.9 mL, 25 mg/L) in methanol was mixed with the sample extracts (0.1 mL), and then the reaction progress was monitored at 515 nm until the absorbance was stable [23].
3. β-Carotene linoleate model system: A mixture of β-carotene (0.2 mg), linoleic acid (200 mg), and Tween-40 (200 mg) was prepared. Chloroform was removed at 40 °C under vacuum. The resulting mixture was diluted with 10 mL of water. To this emulsion was added 40 mL of oxygenated water. Four-milliliter aliquots of the emulsion were added to 0.2 mL of berry extracts (50 and 100 ppm). The absorbance at 470 nm was measured. The AA of the extracts was evaluated in terms of bleaching of the β-carotene: AA = 100 [1 − *(A*_0_ − *A*_t_)/(*A*_0_° − *A*_t_°)], where *A*_0_ and *A*_0_° are the absorbance values measured at zero time of the incubation for test sample and control, respectively, and *A*_t_ and *A*_t_° are the absorbance values measured in the test sample and control, respectively, after incubation for 180 min [24].

### Fluorometric Measurements

Fluorometric measurements were used for the evaluation of the antioxidant activity of berries extracts and their in vitro binding properties to human serum albumin. Two-dimensional (2D-FL) and three-dimensional (3D-FL) fluorescence measurements for all berry extracts at a concentration of 0.01 mg/mL were recorded on a model FP-6500, Jasco spectrofluorometer, serial N261332, Japan, equipped with 1.0 cm quartz cells and a thermostat bath. The 2D-FL was taken at emission wavelengths from 310 to 500 nm and at excitation of 295 nm.

The 3D-FL spectra were collected with subsequent scanning emission spectra from 250 to 500 nm at 1.0-nm increments by varying the excitation wavelength from 200 to 350 nm at 10-nm increments [32]. Quercetin (QUE) was used as a standard. All solutions for protein interaction were prepared in 0.05 mol/l Tris-HCl buffer (pH 7.4), containing 0.1 mol/l NaCl. The final concentration of HSA was 2.0 × 10^−6^ mol/l. The HSA was mixed with quercetin in the proportion HSA/extract = 1:1.

### Statistical Analyses

To verify the statistical significance, mean ± SD of five independent measurements were calculated. Data groups’ distribution character was tested by Shapiro–Wilk normality test and the homogeneity of variance by Levene’s *F* test, both at 0.95 confidence level. Multiple comparisons also known as post hoc tests to compare all possible pairs of means of a group of berries extracts were performed by Student–Newman–Keuls method based on the studentised data range. *P*-values of *<*0*.*05 were considered significant. Linear regressions were also calculated and Pearson correlation coefficients (*R*) were used.

## Results and Discussion

### Bioactive Compounds and Antioxidant Activities

**Fig. 1:**
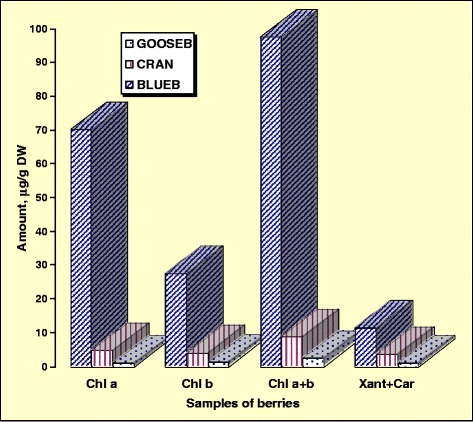
Chlorophyll and carotenoid levels in berries. Values are means ± SD: ±7.15, ±0.48, and ±0.01 for Chl a in BLUEB, CRAN, and GOOSEB, respectively; ±2.45, ±0.43, and ±0.01 for Chl b in BLUEB, CRAN, and GOOSEB, respectively; ±10.08, ±0. 86, and ±0.12 for Chl a + b in BLUEB, CRAN, and GOOSEB, respectively; ±1.25, ±0. 34, and ±0.08 for Xant + Car in BLUEB, CRAN, and GOOSEB, respectively. *Chl* chlorophyll, *Xant* xanthophylls, *car* carotenes, *GOOSEB* gooseberries, *CRAN* cranberries, *BLUEB* blueberries

It was interesting to use different solvent systems such as diethyl ether, ethyl acetate, and water in order to find out the best extraction conditions and the maximum antioxidant activities of gooseberries in comparison with blueberries and cranberries. The results of the determination of the contents of the bioactive compounds in the extracts of three solvents of all studied samples are summarized in the Table 1. As can be seen, the significant highest contents (*P* < 0.05) of polyphenols and flavanols were in the water fraction of blueberries (46.56 ± 4.2 mg GAE/g and 1.75 ± 0.3 mg CE/g, respectively). The contents of flavonoids are comparable with the data in cranberries. The contents of chlorophylls and carotenoids (Fig. 1) were the highest in blueberries as well (*P* < 0.05). The weight ratio of Chl *a* and Chl *b* is an indicator of the functional pigments. The ratios of chlorophylls *a*/*b* were the following: 0.68, 1.17, and 2.55 for gooseberries (GOOSEB), cranberries (CRAN), and blueberries (BLUEB), respectively. The ratio of total chlorophylls to total carotenoids is an indicator of the greenness of plants (Fig. 1).

**Table 1:** Bioactive compounds in water, ethyl acetate, and diethyl ether extracts of gooseberries (*P. peruviana*), cranberries (*V. macrocarpon*), and blueberries (*V. corymbosum*) per gram dry weight

Extracts	Indices		
	POLYPHEN, mg GAE	FLAVON, mg CE	FLAVAN, μg CE
GOOSEB, H_2_O	5.37 ± 0.6	0.22 ± 0.04	nd
CRAN, H_2_O	22.13 ± 2.5	3.83 ± 0.4	467.36 ± 14.5
BLUEB, H_2_O	46.56 ± 4.2	3.89 ± 0.6	1,751.51 ± 25.6
GOOSEB, EtOAc	0.29 ± 0.1	0.11 ± 0.01	nd
CRAN, EtOAc	3.14 ± 0.4	0.66 ± 0.1	44.14 ± 4.3
BLUEB, EtOAc	3.87 ± 0.4	0.74 ± 0.1	112.06 ± 7.4
GOOSEB, DETETHR	0.14 ± 0.01	0.08 ± 0.01	1.21 ± 0.1
CRAN, DETETHR	2.11 ± 0.2	0.10 ± 0.01	7.66 ± 0.8
BLUEB, DETETHR	4.13 ± 0.4	0.39 ± 0.1	32.55 ± 3.9

Values are means ± SD of five measurements. All statistical data are presented in Table 4

*POLYPHEN* polyphenols, *CE* catechin equivalent, *GAE* gallic acid equivalent, *FLAVON* flavonoids, *FLAVAN* flavanols, *nd* not determined, *GOOSEB* gooseberries (*P. peruviana*), *CRAN* cranberries (*V. macrocarpon*), *BLUEB* blueberries (*V. corymbosum*), *EtOAc* ethyl acetate, *DETETHR* diethyl ether

It was mentioned earlier that the main purpose was to compare gooseberry with other berries in order to find out if its bioactivity is on the same level as in other kinds of berry. Therefore, the contents of the bioactive compounds and AA were determined and compared with widely consumed blueberries and cranberries. A number of reviewed articles show that the main bioactive compounds determining the nutritional quality of berries are polyphenols, anthocyanins, and flavonoids [1, 9]. Carotenoids and chlorophylls are important in the composition of berries. The ratio of total chlorophylls to total carotenoids was 2.15, 2.47, and 8.67 for gooseberries, cranberries, and blueberries, respectively. The two ratios were in the range which shows that the berries were grown and collected at optimal growing conditions [33]. The obtained contents of chlorophylls and carotenoids were in acceptable range, showing their sensitivity to seasonal variation in climatic conditions [34]. Our data can be compared with other reports [35], where different carotenoids in seabuck thorn berries increased in concentration during ripening and comprised from 120 to 1,425 μg/g DW of total carotenoids (1.5–18.5 mg/100 g of FW), depending on the cultivar, harvest time, and year. The content of chlorophyll can act as a marker of the degree of ripening.

**Fig. 2:**
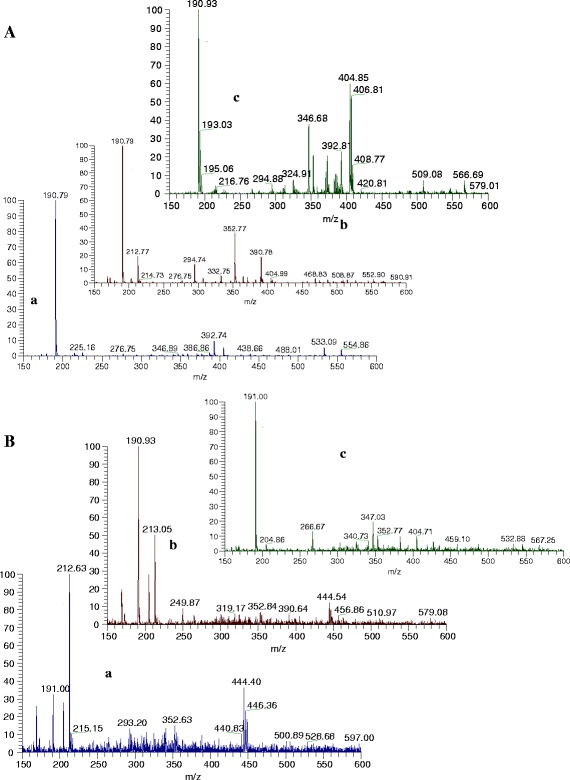

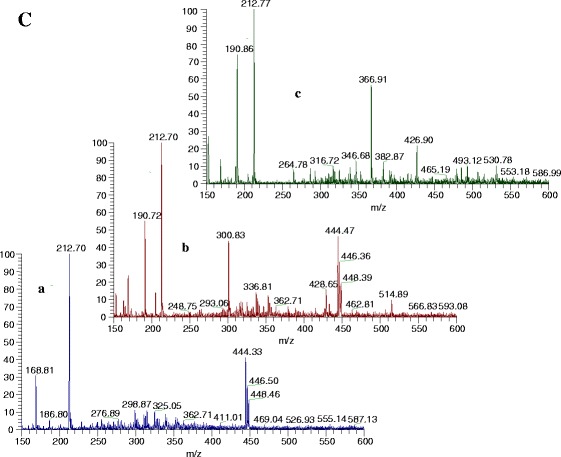
ESI-MS spectra of extracted fractions from three studied berries. **a** Aqueous, **b** ethyl acetate, and **c** diethyl ether of *a* gooseberries, *b* cranberries, and *c* blueberries in negative ion mode. Phenolic compounds were identified at *m*/*z* based on the mass spectra data

We investigated the properties of quercetin, the major phenolic phytochemical present in berries, in aqueous media using UV spectroscopy, fluorometry, and ESI-mass spectrometry. As was declared in “Results and Discussion”, the contents of bioactive compounds (polyphenols, flavonoids, and flavanols) in three different extracts was determined and compared, and the significantly highest amounts were in water extract of blueberries. Gooseberries showed a moderate amount of bioactive compounds. Our results were in agreement with others, showing that water extracts of blueberries contain high amounts of polyphenols [9]. The amount of phenolics for blueberry and cranberry was reported as 261–585 and 315 mg/g FW and for flavonoids as 50 and 157 mg/g FW [36, 37]. The ESI-MS in negative ion mode (Table 2; Fig. 2a) of water extracts differs between berries. The water extract of gooseberry (Table 2; Fig. 2—Aa) showed that the molecular ion at *m*/*z* 190.79 corresponded to quinic acid. Oppositely, cranberry (Table 2; Fig. 2—Ab) water extract was characterized by chlorogenic acid of the [M-H]^−^ deprotonated molecule (*m*/*z* 353) and the ion corresponding to the deprotonated quinic acid (*m*/*z* 191), which was consistent with Sun et al. (2007). Blueberry water extract (Table 2; Fig. 2c) demonstrated a peak at 404.85 (piceatannol 3-*O*-glucoside), 346.68, and 190.93 as a result of destroying 5-heptadecylresorcinol. Ethyl acetate extracts of berries showed similar spectral peaks. Gooseberry (Table 1; Fig. 2—Ba) and cranberry (Table 1; Fig. 2—Bb) were similar in molecular ions but differ in the percentage in MS. Blueberry ethyl acetate extract (Table 2; Fig. 2—Bc) and water extract (Table 2; Fig. 2—Ac) were similar. In the diethyl ether extracts (Table 2; Fig. 2c) of all berries, the main peak was of *m*/*z* 212.6. The spectra of blueberry differ from gooseberry and cranberry with one peak at *m*/*z* 366.9. In gooseberry and cranberry extracts, one common peak appeared at *m*/*z* 444.4, but gooseberry extract is characterized by the peak of gallic acid and in cranberry only quercetin is found.

**Table 2:** Mass spectral data (molecular ion and the major fragment ions of polyphenols extracted from berries)

Extracts	Berries	[M-H]^−^ and fragmentation in ESI, (% in MS)	Compound
Water	Gooseberries	190.79 (100)	Quinic acid
	Cranberries	352.77 (40), 190.79 (100)	Chlorogenic acid, quinic acid
		294.74 (15)	*p*-Coumaroyl tartaric acid
		212.6 (20)	2,3 Dihydroxy-*1*-guaiacyl propanone
	Blueberries	404.85 (60)	Piceatannol 3-*O*-glucoside
		346.68 (40), 190.93 (100)	5-Heptadecylresorcinol, quinic acid
Ethyl acetate	Gooseberries	444.40 (35)	Apigenin 7-*O*-glucuronide
		190.79 (30)	Quinic acid
		212.6 (100)	2,3 Dihydroxy-*1*-guaiacyl propanone
	Cranberries	444.5 (10)	Apigenin 7-*O*-glucuronide
		190.79 (100)	Quinic acid
		212.6 (50)	2,3 Dihydroxy-*1*-guaiacyl propanone
	Blueberries	346.68 (20)	5-Heptadecylresorcinol
		190.79 (100)	Quinic acid
Diethyl ether	Gooseberries	444.33 (40)	Apigenin 7-*O*-glucuronide
		212.6 (100)	2,3 Dihydroxy-*1*-guaiacyl propanone
		168.81 (30)	Gallic acid
	Cranberries	444.47 (40)	Apigenin 7-*O*-glucuronide
		300.83 (40)	quercitin
		212.6 (100)	2,3 Dihydroxy-*1*-guaiacyl Propanone
		190.7 (55)	Quinic acid
	Blueberries	366.9 (50), 190.8 (80)	3-Feruloylquinic acid, quinic acid
		212.7 (100)	2,3 Dihydroxy-*1*-guaiacyl propanone

The recorded spectra were in the same scale (in the range between 100 and 600 *m*/*z*) for comparison. We choose negative mode as the MS method because in many publications it was described that this mode is the best for analysis of low molecular weight phenolic compounds [29, 38–40]. All of the peaks were identified and the recorded MS spectra can be used as a fingerprint for characterization of different berry extracts based on the percentage of the main peaks. Our obtained results by MS are similar to Zuo et al. [39], where 15 benzoic and phenolic acids (benzoic, *o*-hydroxybenzoic, cinnamic, *m*-hydroxybenzoic, *p*-hydroxybenzoic, *p*-hydroxyphenyl acetic, phthalic, 2,3-dihydroxybenzoic, vanillic, *o*-hydroxycinnamic, 2,4-dihydroxybenzoic, *p*-coumaric, ferulic, caffeic, and sinapic acid) were identified in cranberry fruit. The most abundant is benzoic and then *p*-coumaric and sinapic acids. The phenolic constituents in the berries were identified as chlorogenic acid, *p*-coumaric acid, hyperoside, quercetin-3-*O*-glucoside, isoorientin, isovitexin, orientin, and vitexin [38]. The AA of blueberry in water extracts (Table 3) as determined by CUPRAC, DPPH, and β-carotene assays (131.09 ± 12.9.3 μM TE/g DW, 108.09 ± 7.2 μM TE/g DW, and 80.11 ± 8.9 %, respectively) in all of the extracts used is significantly higher than that recorded for other berries studied (*P* < 0.05). The AA of gooseberry is lower by about nine times than in blueberries and four times than in cranberries. As was calculated, a very good correlation was found between the antioxidant activity and the contents of total polyphenols in water extracts. All groups of data (Tables 1 and 3) were tested for character of their distribution and homogeneity of variance at 0.95 confidence level. The Shapiro–Wilk normality test showed that all the data in groups are normally distributed, with the exception of flavanols in gooseberry water and ethyl acetate extracts with no quantified content. Levene’s F test, which is widely accepted as the most powerful homogeneity of variance test, indicated extract types which have no the same variance tested at 0.95 confidence level. Table 4 presents significant differences (with *P* values *<*0*.*05) between bioactive compounds contents and antioxidant activities in different extracts of berries found by multiple comparisons using the method of Student–Newman–Keuls. The method denotes significantly different pairs, and the group in the first position means that it is higher in the contents of bioactive substances. For example, the case of polyphenols in line G/W-G/D means a statistically different content of polyphenols between gooseberry water and diethyl ether extracts. Water extract is higher in the content of polyphenols of about 10.2 mg GAE/g DW. From Table 4, it is evident that in majority of the cases, water extraction yields the highest content of bioactive compounds and antioxidant activities.

**Table 3:** Antioxidant activities in water, ethyl acetate, and diethyl ether extracts of gooseberries (*P. peruviana*), cranberries (*V. macrocarpon*), and blueberries (*V. corymbosum*) per gram dry weight

Extracts	Indices		
	DPPH, μM TE/g DW	CUPRAC, μM TE/g DW	β-carotene, %
GOOSEB, H_2_O	8.39 ± 0.9	11.25 ± 1.1	11.40 ± 0.9
CRAN, H_2_O	46.58 ± 4.5	49.38 ± 4.4	36.71 ± 3.8
BLUEB, H_2_O	108.09 ± 7.2	131.09 ± 9.6	80.10 ± 6.6
GOOSEB, EtOAc	0.35 ± 0.1	0.88 ± 0.1	0.54 ± 0.1
CRAN, EtOAc	3.02 ± 0.4	9.20 ± 1.1	6.09 ± 0.6
BLUEB, EtOAc	8.83 ± 4.4	12.40 ± 1.1	8.13 ± 0.9
GOOSEB, DETETHR	0.16 ± 0.01	0.24 ± 0.01	0.20 ± 0.01
CRAN, DETETHR	3.42 ± 0.4	5.77 ± 0.6	3.48 ± 0.3
BLUEB, DETETHR	10.97 ± 0.9	14.87 ± 1.1	6.79 ± 0.7

Values are means ± SD of five measurements. All statistical data are shown in Table 4

*DW* dry weight, *DPPH* 2,2-diphenyl-*1*-picrylhydrazyl, *CUPRAC* cupric reducing antioxidant capacity, *β-carotene* β-carotene linoleate assay, *GOOSEB* gooseberries (*P. peruviana*), *CRAN* cranberries (*V. macrocarpon*), *BLUEB* blueberries (*V. corymbosum*), *EtOAc* ethyl acetate, *DETETHR* diethyl ether

**Table 4:** Statistically significant differences between the content of bioactive compounds in different extracts of berries by Student–Newman–Keuls multiple comparisons

Comparison between berries extracts	Difference	Standard error	*q* stat	Table *q*	Probability, *P <* 0*.*05
Polyphenols					
G/W–G/D	10.2053	0.7071	14.4325	3.6332	0.0000
G/E–G/D	8.6337	0.7071	12.2099	2.7718	0.0000
B/W–B/D	4.3603	0.7071	6.1665	3.6332	0.0001
B/W–B/E	3.8084	0.7071	5.3860	3.3145	0.0004
Flavonoids					
G/W–G/E	2.7948	0.7071	3.9525	3.6332	0.0267
C/W–C/D	7.0963	0.7071	10.0357	4.0301	0.0000
C/E–C/D	4.3453	0.7071	6.1452	3.8577	0.0001
B/W–B/E	4.1482	0.7071	5.8665	3.8577	0.0003
B/W–B/D	4.1482	0.7071	5.8665	3.6332	0.0002
C/W–C/E	2.7510	0.7071	3.8905	2.7718	0.0059
Flavanols					
G/W–G/D	3.2040	0.7071	4.5311	3.3145	0.0039
G/E–G/D	3.2040	0.7071	4.5311	2.7718	0.0014
C/W–C/D	6.3189	0.7071	8.9363	3.8577	0.0000
C/E–C/D	3.9136	0.7071	5.5347	3.3145	0.0003
B/W–B/D	4.6555	0.7071	6.5839	3.8577	0.0000
C/W–C/E	2.4053	0.7071	3.4016	3.3145	0.0427
B/W–B/E	2.7159	0.7071	3.8409	3.3145	0.0181
DPPH					
G/W–G/D	12.0877	0.7071	17.0946	4.0301	0.0000
G/E–G/D	7.8126	0.7071	11.0486	2.7718	0.0000
G/W–G/E	4.2751	0.7071	6.0460	3.8577	0.0002
C/W–C/E	4.3824	0.7071	6.1976	4.0301	0.0002
C/W–C/D	4.3289	0.7071	6.1219	3.8577	0.0001
B/W–B/D	4.2085	0.7071	5.9517	3.8577	0.0002
B/W–B/E	2.8095	0.7071	3.9733	3.3145	0.0138
CUPRAC					
G/W–G/D	9.7648	0.7071	13.8095	4.0301	0.0000
G/E–G/D	4.4785	0.7071	6.3335	2.7718	0.0000
G/W–G/E	5.2863	0.7071	7.4760	3.8577	0.0000
C/W–C/D	4.8131	0.7071	6.8068	4.0301	0.0000
B/W–B/E	4.3484	0.7071	6.1495	4.0301	0.0002
B/W–B/D	4.1359	0.7071	5.8490	3.8577	0.0003
C/W–C/E	2.9609	0.7071	4.1874	2.7718	0.0031
β-CAROTENE					
G/W–G/D	8.5379	0.7071	12.0744	4.0301	0.0000
G/E–G/D	3.8783	0.7071	5.4847	2.7718	0.0001
G/W–G/E	4.6596	0.7071	6.5897	3.8577	0.0000
C/W–C/D	5.2270	0.7071	7.3921	4.0301	0.0000
C/W–C/E	3.6094	0.7071	5.1045	3.8577	0.0028
B/W–B/D	4.0614	0.7071	5.7437	3.8577	0.0005

*B* berries, *G* gooseberries, *C* cranberries, *B* blueberries, *W* water, *E* ethyl acetate, *D* diethyl ether

The antioxidant activity of different extracts was evaluated by DPPH free radical scavenging activity, taking total phenolic content as an index [41]. Our obtained results correspond with the data of Kusznierewicz et al. [17], where the DPPH antioxidant activity varied from 93 to 166 mol TE/g DW. The obtained phenolic compounds and DPPH values (Tables 1 and 2) were as well in the range of those reported by Li et al. [42] of four berry fruits (strawberry, Saskatoon berry, raspberry, and wild blueberry), chokecherry, and seabuck thorn ranging from 22.83 to 131.88 g/kg and DPPH ranging from 29.97 to 78.86 %. The bioactivity of blueberry is significantly higher than the bioactivity of other berries; however, this index in the gooseberry is comparable with the studied samples. According to the results of Table 4, the antioxidant activities of extracts, partitions, and fractions were strongly correlated with the highest polyphenol contents. Correlation between polyphenols and antioxidant properties exactly corresponded with our results: the highest phenolic content was found in walnut, which revealed the best antioxidant properties [43]. This corresponds with Seeram [8], who discussed also that phytonutrients ranged from fat-soluble/lipophilic to water-soluble/hydrophilic compounds. Our results about the high antioxidant activity of berries (Table 3) are in line with Elberry et al. [11], showing a high antioxidant activity of cranberry extract. Pronounced antioxidant and radical scavenging properties of cranberry was shown by Wojnicz et al. [12]. Ethanol-soluble acidic components were used in order to determine the bioactivity of natural novel sources against oxidation [44]. Our results are in accordance with You et al. [45], where four Rabbiteye blueberry cultivars grown organically and conventionally were compared by their total phenolic content and antioxidant values by DPPH and CUPRAC. Our studies are not in full correspondence with others [15] based on the different extraction systems. In our case, the most active was the water fraction of *P. peruviana* (PP) in comparison with ethyl acetate and diethyl ether. As was reported by Wu et al. [15], supercritical carbon dioxide SCEPP-5 PP extracts in comparison with hot water and ethanol possessed the highest total flavonoid (226.19 mg/g) and phenol (100.82 mg/g) contents. Our results connected with other reports [41, 46], where the methanol extract of leaves from some plants was more potent against *Aspergillus fumigatus* and *Candida tropicana.* The lowest MIC values obtained for LM, LA, and LH were 78, 156, and 625 μg/mL against *A. fumigatus*, *C. tropicana*, and *C. albicans*, respectively [41]. Our results correspond as well with Suwalsky et al. [19], who showed a new kind of Chilean berries, and the polyphenol aqueous extracts of leaves and whole fruit were responsible for the antioxidant properties when the extracts were induced to interact with human red cells. The results of the CUPRAC test showed that cranberry juice had the highest level of antioxidant reactivity, blueberry juice had an intermediate activity, and orange juice had the lowest. It was determined, however, that contrary to the hypothesis, orange juice was significantly more potent in protecting the bladder against ischemia/reperfusion damage than either blueberry or cranberry juice. Thus, it is concluded that chemical tests for TAA do not necessarily correlate with their physiological activity [2]. The obtained antioxidant activity by FRAP of blueberry and cranberry extracts was similar to other studies. Probably, a complex spectrum of anthocyanins was the major contributor to the antioxidant activity [47].

### Fluorometry Spectra Studies

**Fig. 3:**
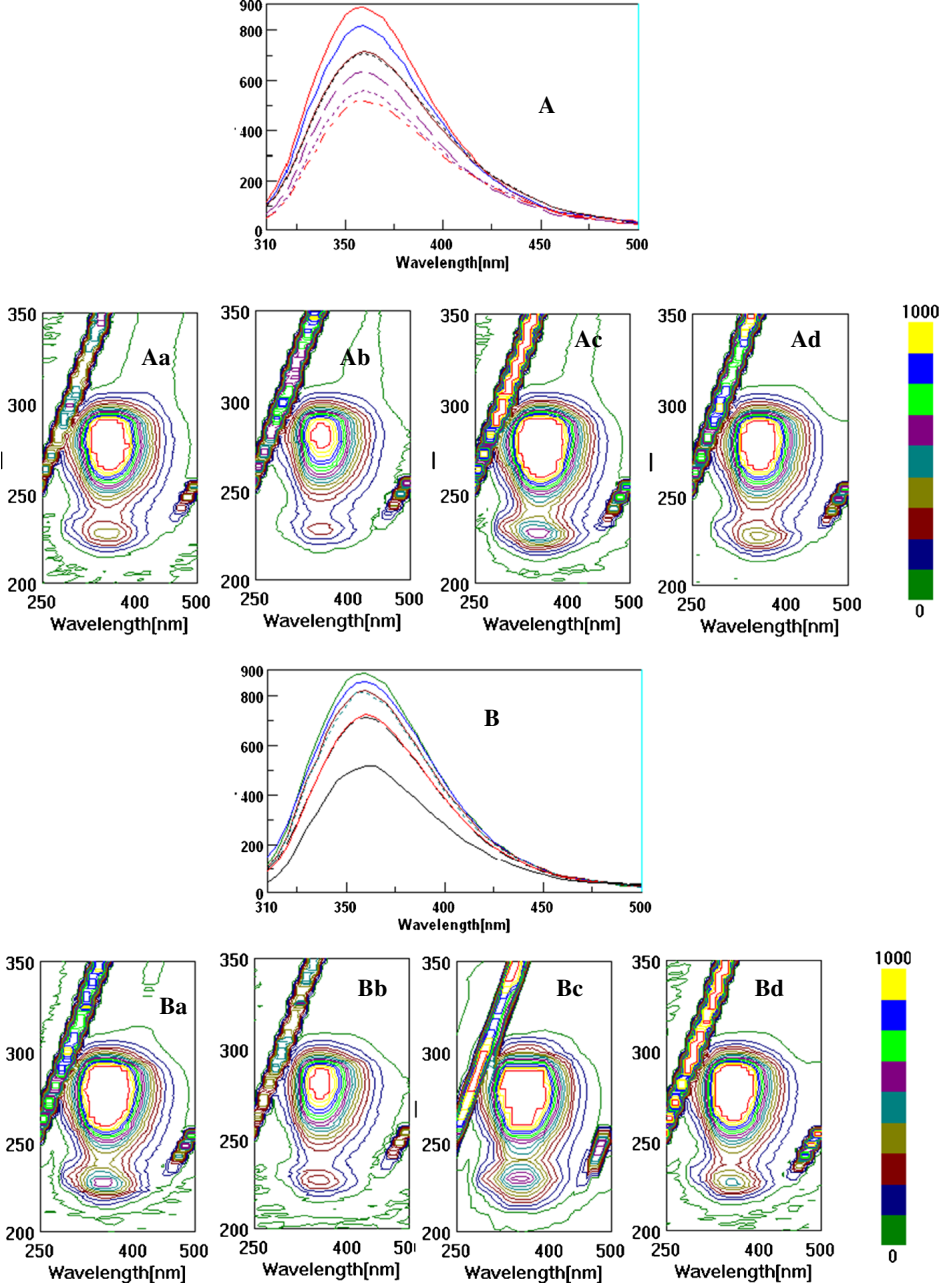
Two-dimensional fluorescence (2D-FL) and three (3D-FL) spectra illustrate the interaction between HSA, quercetin, aqueous (positions *Aa*, *Ab*, *Ac*, and *Ad*), and ethyl acetate (positions *Ba*, *Bb*, *Bc*, and *Bd*) extracts of studied berries. **a** Change in the fluorescence intensity as a result of binding affinity with water extracts: HSA [*first line* from the *top* with FI of 890.21], HSA + WGOOSEB (*second line* from the *top* with FI = 817.50), HSA + WCRAN (*third line*, FI = 717.39), HSA + WBLUEB (*fourth line*, FI = 709.75), HSA + WGOOSEB + QUE (*fifth line*, FI = 635.24), HSA + WCRAN + QUE (*sixth line*, FI = 560.83), and HSA + WBLUEB + QUE (seventh line, FI = 518.96). *Aa*–*Ad* cross maps from the 3D-FL spectrum of HSA + WBLUEB, HSA + WBLUEB + QUE, HSA + WGOOSEB, and HSA + WGOOSEB + QUE. **b** Change in the fluorescence intensity as a result of binding affinity of HSA with ethyl acetate extracts: HSA [*first line* from the *top* with FI of 890.21], HSA + EtOAcGOOSEB (*second line*, FI = 834.70), HSA + EtOAcCRAN (*third line*, FI = 821.65), HSA + EtOAcBLUEB (*fourth line*, FI = 811.70), HSA + EtOAcGOOSEB + QUE (*fifth line*, FI = 724.76), HSA + EtOAcCRAN + QUE (*sixth line*, FI = 713.41), and HSA + EtOAcBLUEB + QUE (*seventh line*, FI = 618.96). *Ba*–*Bd* cross maps from the 3D-FL spectrum of HSA + EtOAcBLUEB, HSA + EtOAcBLUEB + QUE, HSA + EtOAcGOOSEB, and HSA + EtOAcGOOSEB + QUE. In all reactions, the following conditions were used: HSA (2.0 × 10^−6^ mol/L), quercetin (1.7 × 10^−6^ mol/L), and water and EtOAc extracts in concentration of 25 and 50 μg/ml, respectively. Binding was during 1 h at 25 °C. Fluorescence intensities are on *y*-axis and emission wavelengths are on *x*-axis. *HSA* human serum albumin, *QUE* quercetin, *EtOAc* ethyl acetate, *WGOOSEB* water extracts of gooseberry, *WCRAN* water extracts of cranberry, *WBLUEB* water extracts of blueberry, *EtOAcGOOSEB* ethyl acetate extracts of gooseberry, *EtOAcCRAN* ethyl acetate extracts of cranberry, *EtOAcBLUEB* ethyl acetate extracts of blueberry

**Fig. 4:**
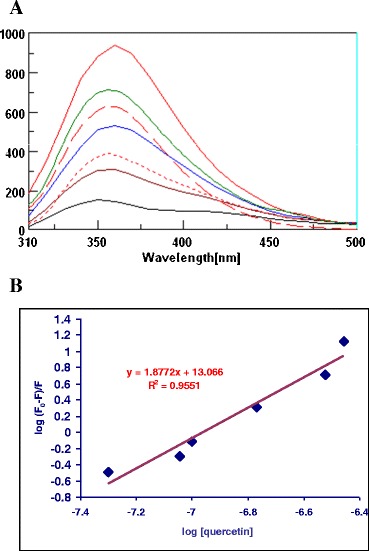
**a** Fluorescence spectra of aqueous solutions of HSA (2.0 × 10^−6^ mol/L) in the presence of different concentrations of quercetin: 0, 0.17, 0.30, 1.0, and 1.7 × 10^−6^) mol/L at pH 7.4 at excitation wavelength of 290 nm. **b** Linear plot for log (*F*_0_ − *F*)/*F* vs log [quercetin], where *F*_0_ and *F* represent the fluorescence intensity of HSA in the absence and in the presence of polyphenols, respectively

The binding properties of the berry samples in comparison with the pure flavonoids such as quercetin are shown in 3D- FL spectra, which illustrated the elliptical shape of the cross map. The results showed that the 3D- FL cross maps of berries differed. One of the main peaks for HSA was found at *λ*ex/em of 220/360 nm. The second main peak appeared for these samples at *λ*ex/em of 280/350 nm (Fig. 3a, b). The interaction of HSA and the water and ethyl acetate extracts of berries (Fig. 3—Aa, Ac, Ba, and Bc), HSA, water, and ethyl acetate extracts, and quercetin (Fig. 3—Ab, Ad, Bb, and Bd) showed a slight change in the position of the main peak at the wavelength of 360 nm and a decrease in fluorescence intensity (FI). The following changes appeared when the water extracts of berries were added to HSA [initially the main peak at emission 360 nm and FI of 890.21] (Figs. 3a, b and 4a, b; the upper line is HSA). The reaction with the berry extracts and quercetin decreased the FI of HSA (Fig. 3a, b; middle and low lines). The following decrease in the FI (%) occurred during the interaction of water extracts with HSA: HSA + WGOOSEB = 8, HSA + WCRAN = 19.4, and HSA + WBLUEB = 20.3. The decrease in the FI with ethyl acetate extracts was lower than with water extract: HSA + EtOAcGOOSEB = 6.0, HSA + EtOAcCRAN = 7.7, and HSA + EtOAcBLUEB = 8.2. The diethyl ether extracts did not show any binding properties with HSA. These results are in direct relationship with the antioxidant properties of the extracts. The synergism of bioactive compounds is shown when quercetin was added to the mixture of HSA and extracts of berries. The decrease in the FI of HSA with WGOOSEB, WCRAN, and WBLUEB was 28.6, 37.0, and 41.7, respectively (fifth, sixth, and seventh lines (Fig. 3a)). Therefore, the participation of quercetin in synergism was 20.6, 17.6, and 21.4 for WGOOSEB, WCRAN, and WBLUEB, respectively. With ethyl acetate extracts, the participation of quercetin was 13.9, 10.9, and 17.6 for GOOSEB, CRAN, and BLUEB, respectively (Fig. 3b).

The concentrations of water extracts of berries in the interaction with HSA are 3.01971, 5.12232, and 5.23493 × 10^−8^ QUE for GOOSEB, CRAN, and BLUEB, respectively. Ethyl acetate extracts showed lower concentrations at 2.5751, 2.90949, and 3.16139 × 10^−8^ for GOOSEB, CRAN, and BLUEB, respectively. Our very recent results showed that the fluorescence is significantly quenched because the conformation of the HSA changes in the presence of pure flavonoids and berry extracts. This interaction between quercetin and HSA was investigated using tryptophan fluorescence quenching. Our result is in agreement with others that quercetin, as an aglycon, is more hydrophobic and demonstrates strong affinity toward HSA. Other results [20, 21] differ from those reported by us, probably because of the variety of antioxidant abilities of pure flavonoids and different ranges of fluorometry scanning ranges used in a similar study. The biological relevance of quercetin interaction in human organism is important from the point of view that this molecule of polyphenolic type extensively binds to HSA, the most abundant carrier protein in the blood. Our in vitro results of interaction of HSA and quercetin can be compared with other reports in vivo, showing the protective effects of quercetin on hepatic injury induced by different chemical reactions. Our results on BSA binding with other types of berry correspond with our present results with HSA and investigated berries. Results on water extracts of blueberries were similar to these samples [48, 49]. Strong binding properties have been confirmed for the compounds containing high bioactivity. The strong binding properties of phenolics show that they may be effective in the prevention of atherosclerosis under physiological conditions. Quercetin can suppress HSA. These results demonstrate that quercetin and other phenolic compounds can effectively protect from atherosclerosis under physiologically relevant conditions, providing insight into the mechanism of action of bioactive phenolics. Our explanation of the binding affinity of berry polyphenols is similar to the description of Xiao et al. [20] and Xiao and Kai [21] that one or more hydroxyl groups in the B-ring of flavonoids enhanced the binding affinities to proteins. Much of the bioactivities of citrus flavanones significantly appear to impact blood and microvascular endothelial cells [50]; therefore, it was essential to investigate the interaction between berry polyphenols and serum albumin. The binding constants ranked in the following order: quercetin>rutin>calycosin>calycosin-7-*O*-(sup)-*D*-glucoside [formononetin-7-*O*-(sup)-*D*-glucoside [51]. 3D fluorescence can be used as an additional tool for the characterization of the polyphenol extracts of berry cultivars and their binding properties.

## Conclusion

There are many reports on the antioxidant properties of berries; however, there is little information about the binding properties of blueberries and cranberries and even less information about gooseberries. The gooseberry, in comparison with cranberries and blueberries, showed a lower amount of bioactive compounds. Therefore, some of the methods used in this work such as fluorescence were done for the first time. Some of the active compounds may have synergistic interactions with other compounds as was shown when quercetin was added to the reaction. This work demonstrated relatively high antioxidant and binding properties of the investigated berries, especially in water extracts. The possibility of benefit of the consumption of these berries for everyday human health can be suggested.
